# Mental images, entrapment and affect in young adults meeting criteria of nonsuicidal self-injury disorder (NSSID) – a daily diary study

**DOI:** 10.1186/s40479-019-0117-0

**Published:** 2020-02-12

**Authors:** Marie Cloos, Martina Di Simplicio, Florian Hammerle, Regina Steil

**Affiliations:** 1 Department of Clinical Psychology and Psychotherapy, Institute of Psychology, Goethe University, Frankfurt Main, Germany; 2 Division of Psychiatry, Department of Brain Sciences, Imperial College London, London, UK; 3 Department of Child and Adolescent Psychiatry and Psychotherapy, University Medical Center of the Johannes Gutenberg-University, Mainz, Germany

**Keywords:** NSSI, NSSID, Mental images, NSSI-images, Daily diary

## Abstract

**Background:**

Incidents of nonsuicidal self-injury (NSSI) are often accompanied by mental images which could be perceived as distressing and/or soothing; yet existing data is derived from participants with a history of NSSI using retrospective methods. This study investigated mental images related to NSSI (“NSSI-images”), and their relationship to the proposed Nonsuicidal Self-Injury Disorder (NSSID).

**Methods:**

An e-mail was sent to all female students of the local University providing the link to an online screening and 201 students with a history of repetitive NSSI responded. Nineteen eligible participants meeting criteria of NSSID (mean age = 25; 32% with migrant background) further completed a baseline interview and a ten-day-diary protocol.

**Results:**

Among the sample of *N* = 201, 83.6% reported NSSI-images. In the subsample of *n* = 19 diagnosed with NSSID, the frequencies of NSSI and NSSI-images were correlated; about 80% of the most significant NSSI-images were either of NSSI or of an instrument associated with NSSI (i.e., a razorblade). In the diary, 53% of the sample self-injured. NSSI-images were reported on 94% of NSSI-days, and on days with NSSI and NSSI-images, the images almost always occurred first; the images were overall perceived as twice more distressing than comforting. Images on NSSI-days were characterized by more comfort, intrusiveness and compellingness yet less vividness, and increased subsequent positive and negative affect compared to non-NSSI days. NSSI-days were further marked by increased entrapment beliefs and increased negative yet decreased positive affect at night. These results were non-significant.

**Limitations:**

Due to non-significant results among a small sample size and a low rate of NSSI among the NSSID-group, results remain preliminary.

**Conclusions:**

The study provides information on feasibility and methodological challenges such as intervention effects of the diary. NSSI-images may be common among individuals who engage in NSSI; they may capture ambivalent (positive and negative) appraisals of NSSI and thus play a role in NSSI and possibly a disorder such as NSSID. The preoccupation with NSSI (Criterion C of NSSID in DSM-5) may as well be imagery-based.

**Registration:**

The study was retrospectively registered with the DRKS under the number DRKS00011854.

## Introduction

Nonsuicidal self-injury (NSSI) is defined as “the deliberate, self-inflicted damage of body tissue without suicidal intent and for purposes not socially or culturally sanctioned” [1] such as cutting or burning of the skin. NSSI is present among 13.4% of young adults [2] and tends to decline over the life span [3]. The behavior may be associated with serious impairments [4], among them an elevated risk for completed suicide [5].

Nonsuicidal self-injury disorder (NSSID) is listed in the fifth version of the Diagnostic and Statistical Manual of Mental Disorders (DSM-5 [6])^1^ and has not been sufficiently evaluated yet according to some [7]. The 11th revision of the International Classification of Diseases (ICD-11) lists no separate disorder concerning NSSI [8]. Some studies have provided support for the validity of NSSID, e.g., for the positive and negative reinforcement mechanisms (for a review, see [9]) or the proposed minimum of five or more incidents during the past 12 months ([10]; while other data suggests + 10 incidents might be more valid [11]) and the use of the criteria may improve the assessment of the disorder [9]. However, unresolved issues include the delimination from other psychiatric conditions [12], and the controversial debate on, e.g., criterion E regarding distress/ impairment because NSSI can also be appraised as helpful [13] and not necessarily as causing intense distress [14] by people who engage in it (for a review, see [9]).

NSSI can be a symptom of Borderline Personality Disorder (BPD [6]), and distinction between both helps validate a potential diagnosis of NSSID [15]. Concurrent BPD diagnoses were found in 51.7% [16] and as little as 20.5% [17] respectively of young adolescents diagnosed with NSSID which rather supports a separate diagnosis [7]. Around half of those who had shown NSSI during adolescence met the diagnosic criteria of BPD in young adulthood and earlier onset of NSSI was a risk factor for a later BPD diagnosis [18].

According to the proposed criteria in the DSM-5, NSSI can be maintained by negative and positive reinforcement mechanisms (for a review, see [9]): NSSI-incidents are preceded by interpersonal problems or negative emotional states and carried out with the expectation to thereby ameliorate such problems (negative reinforcement [6]). The positive reinforcement or appetitive aspects of NSSI are characterized by frequent thoughts about NSSI, strong urges to self-injure, and the expectation that NSSI may bring about positive emotions [6]. Accordingly, a recent study has linked elevated levels of positive emotions in combination with low levels of negative emotions to an increased NSSI frequency [19].

In conclusion, there are high prevalence rates of NSSI in young adults apart from the diagnosis of BPD, and diagnostic features of NSSID remain discussed. There seems to be a need for further research to understand NSSI and its phenomenology, and in particular if this could better inform the definition of NSSID.

The current study attempts to shed light on the proposed characteristics of NSSID as they relate to mental imagery, or images 'in the mind’s eye' [20]. Both the negative and the positive aspects of NSSI may be accompanied by mental images as imagery-based thinking can amplify emotional processes [21]. Mental images are memory-based mental events that can involve all five senses although some concepts prioritize the visual quality [22]. They can occur without any current sensory input [23] and can be distinguished from more symbolic ways of thinking such as verbal thoughts [24]. Mental images can be associated with activity in brain-regions that are active during actual perceptions (e.g., [25]) and can thus feel very real (e.g., [26]). Imagination and fantasy can be part of healthy psychological functioning [27] yet psychological disorders are often accompanied by strongly valenced and distressing imagery, e.g., flashbacks in PTSD (e.g., [28]). Mental imagery in psychopathology can be of distressing and negative nature, e.g. after a traumatic experience in PTSD (e.g., [28]) or an actual or feared loss in depression (e.g., [29]), yet has also been perceived as emotionally ambivalent or even positive such as imagery of desired objects at times of craving for substances (e.g., [30]) or imagery of escape after-life scenarios associated with suicidal ideation (e.g., [31]). Mental imagery can contribute to mood swings, e.g. in the bipolar phenotype [32], and to mood lability across disorders [33]. Mental images can also evoke strong motivational responses [30], encapsulating desires and cravings to engage in both adaptive [34] and maladaptive [35] behavior.

In a community survey, 95% of individuals who were prone to dysregulated behaviors including NSSI endorsed negative mental images [26]. Individuals who self-injured have reported to mentally re-experience episodes of self-injury [36], and more than 90% stated that their cognitions prior to acts of NSSI were at least partly imagery-based [37]. These findings suggest that NSSI-related mental images (NSSI-images) may contribute to NSSI and that the frequency of NSSI-images may relate to the frequency of NSSI, yet research on such images is still scarce and to our knowledge mostly derived from either qualitative, or questionnaire-based retrospective studies.

### Characteristics of NSSI-images

NSSI-images have been broadly identified as images that are subjectively associated with NSSI, including (but not exclusively) images of actual NSSI. Studies based on retrospective accounts of people with a history of NSSI report that certain characteristics of NSSI-images may be associated with increased or decreased likelihood for subsequent NSSI. Characteristics may refer to content (NSSI-images preceding NSSI-incidents were mostly of the anticipated injury while images during urges that did not result in NSSI were more likely to be of negative impact of NSSI [37]), intrusiveness and distress (while intrusive and distressing NSSI-images could be associated with subsequent acts of NSSI; deliberately evoked images could seemingly serve as a substitute for NSSI; [36]). Imagery distress in general has been related to the intrusiveness of images across psychopathology [24]. Further, NSSI-images that preceded acts of NSSI could be associated with both distressing (self-critical, hopeless) thoughts and comforting thoughts about emotional relief [37]. Thus, NSSI may be reinforced by both distressing and comforting imagery-based cognitions despite their seeming contradiction. Distressing and compelling mental images of own future suicide have been reported by individuals with a history of suicidal ideation and termed ‘flashforwards’, e.g., ‘*Standing on a wall of a bridge. Imagining jumping off and drowning.*’ [38]. These findings may be potentially relevant to NSSI-images too, given similarities between suicidal and self-injurious behavior. The presence of flashforwards may indicate current suicidal ideation, which declined as flashforwards reduced [31]. Flashforwards were typically rich in sensory detail and perceived as particularly compelling and realistic with a high sense of ‘nowness’ [39] and appraised as positive by at least a third of suicidal individuals [31]. While the perceived comfort of suicidal flashforwards was associated with lower imagery distress in one study [40], Holmes et al. [39] found again highly ambivalent appraisals (both high distress and comfort) in some individuals. Importantly, suicidal images are considered among the factors that facilitate the transition from a motivational to a volitional phase in suicidal behavior [41].

#### NSSI-imagery in BPD

A recent study reported that suicide-related mental imagery occurs in BPD and is associated with suicidal ideation; in particular, patients with BPD and comorbid PTSD reported significantly more vivid images than patients with major depressive disorder [83]. Moreover, a script-driven imagination of an episode of NSSI has been shown to decrease negative affect [42], and FMRI-data has shown that compared to healthy controls, patients with BPD could have altered activation patterns in the brain during imagination of NSSI that were, e.g., associated with diminished emotion regulation and impulse control [43]. Reductions in BPD-symptoms were reported after imagery-based psychotherapeutic interventions during schema therapy such as imagery rescripting (for a review, see [44]).

### Entrapment and negative affect

Flash-forwards in the context of suicidal ideation have also related to perceptions of entrapment [31]. Entrapment or arrested flight occurs when individuals feel defeated and wish to escape their current situation, yet subjectively lack the means to do so [45]. The combination of defeat and entrapment beliefs has been hypothesized as a mechanism explaining suicidal behavior [46]. Entrapment has also been inversely related to self-efficacy [47], which in turn was a protective factor against the emergence of NSSI [48]. However, no study has directly investigated the relationship between entrapment beliefs and NSSI yet.

Finally, another factor that can play a role in NSSI is intense negative affect [49], which has been associated with increased odds for NSSI [19, 50]. Many studies found a decrease of negative affect after NSSI (e.g., [51]), partly in combination with increased negative affect prior to NSSI [52]; yet negative affect may also increase following NSSI [53]. Positive affect tended to decrease before and increase after acts of NSSI [54].

### Aims and hypotheses

The first aim of the current study was to assess the rate of NSSI-imagery among young adults who have repetitively engaged in NSSI using an online screening. We define NSSI-images as images that are subjectively associated with NSSI, including (but not exclusively) images of actual NSSI. The second aim was to investigate the relationship between NSSI and NSSI-imagery in participants meeting the proposed DSM-5 diagnosis of NSSID, both retrospectively in an interview and in a daily diary. To assess this, we compared presence and characteristics of NSSI-images, and associated affect and beliefs on days when NSSI was carried out (NSSI-days) and days when NSSI was not carried out. We hypothesized that the frequency of NSSI would be positively associated with the frequency of NSSI-images. In terms of imagery characteristics, affect and beliefs, we hypothesized that NSSI-images would be more comforting and distressing, less controllable, more compelling, and have greater ‘nowness’ (i.e., the picture or scene in the image would be perceived as happening here and now) on NSSI-days compared to NSSI-images on days without NSSI; levels of entrapment and of negative affect would be higher on NSSI-days than on days without NSSI.

## Methods

### Procedure and sample selection

The study contained three parts: an online-screening, a baseline assessment (including an interview and questionnaires), and an online diary over the course of 10 days. The screening and the diary assessments were programmed online using Unipark (Questback GmbH), a software provider for online surveys. Informed consent was obtained prior to all parts of the study. Ethical approval was obtained from the ethics committee of the psychological faculty of the Goethe University of Frankfurt. The study was registered at the German Clinical Trials Register under the registry number DRKS00011854.

**Fig. 1:**
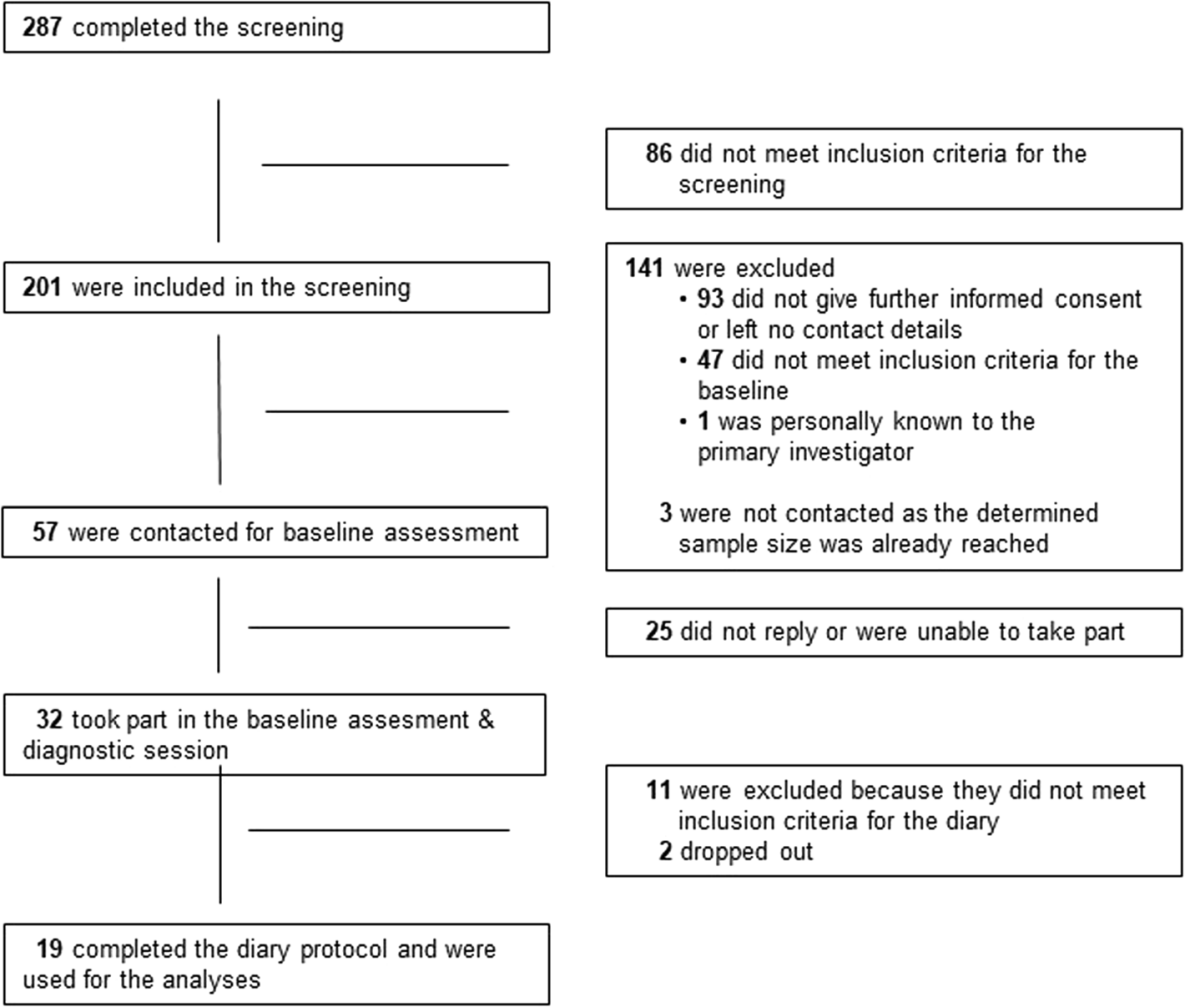
Flowchart of study participants. Displayed are the numbers of participants who were administered into and remained in the study (left side) and the numbers of participants who left the study and the respective reasons for this (right side)

**Fig. 2:**
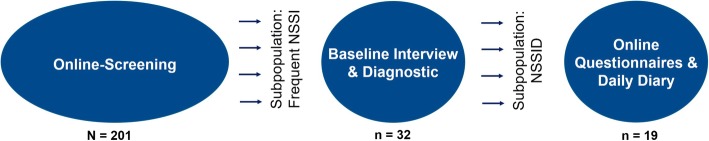
Flow of procedures and final number of participants in the respective parts

Prior to data collection, one pilot participant completed all three parts to ensure feasibility. Figure 1 displays the flowchart of participants, and Fig. 2 displays the flow of procedures and the numbers of participants in the parts of the study.

Online-Screening Procedure (part I of sampling): All female students of the Goethe University of Frankfurt received an e-mail that provided the link to the online screening and invited them to take part in the study if they had engaged in NSSI five or more times during the previous 12 month-period (A-criterion of NSSID in the DSM-5 [6]). The link was further disseminated via course-specific e-mail listings and Facebook groups. Female participants were chosen to reduce the variance in our sample, as previous research has shown that NSSI can vary significantly by sex (e.g., [55]). *Inclusion criteria* for the screening were thus the A-criterion of NSSID, self-identification as female and as a student. Participants of the screening could leave a contact address in case they were interested to take part in further parts of the study.

Baseline Assessment (part II of sampling): *Inclusion criteria* for the baseline assessment were engagement in NSSI at least once during the week before the screening as well as any experience with NSSI-images during the month before the screening.

Daily Diary (part III of sampling): The *inclusion criterion* for the diary was the diagnosis of NSSID as proposed in the DSM-5. Criterion A is defined as engagement in NSSI on 5 or more days in the past year. Criterion B refers to the expectation that NSSI will solve an interpersonal problem, provide relief from unpleasant thoughts and/or emotions, or induce a positive emotional state. Criterion C concerns the experience of one or more of the following: (a) interpersonal problems or negative thoughts or emotions immediately prior to NSSI, (b) preoccupation with NSSI that is difficult to manage, or (c) frequent thoughts about NSSI. Criterion D describes that the NSSI is not socially sanctioned or restricted to minor self-injurious behaviors. Criterion E includes that NSSI is related to clinically significant distress or interference across different domains of functioning (e.g., work, relationships). Criterion F states that NSSI does not occur only in the context of psychosis, delirium, or substance use/withdrawal and is not better accounted for by another psychiatric disorder or medical condition. *Exclusion criteria* for the diary were acute suicidal and homicidal risks, schizophrenia and associated disorders, and any substance dependences (according to the German version of the structured clinical interview; SKID I and II [56]).

Participants from the screening were contacted and invited until all *n* = 19 had completed all parts of the study. We further checked that this number (*n* = 19) would provide sufficient power to detect a large effect size (d = .81; α = 0.05; 1-β = 0.95 [57]) that we assumed based on a previous report by Weßlau et al. [84].

During the diary protocol, participants were provided daily with a link for the questionnaires. To integrate the diary into the daily routine, participants were instructed to fill in the questionnaire at around the same time every night yet were given time until the next day if an entry had been missed. If participants received any psychopharmacological treatment, they were asked to keep it stable over the course of the study.

A random code was given to the participants at baseline for all online material to grant anonymity. As incentives, three vouchers for an online store of 15€ each were raffled among the participants of the screening; credits for students were given for each part if desired; time for completion of all parts was compensated with 30€.^2^ Participants were provided with the contact address of the first author and were encouraged to reach out should they have questions or need any help. All participants who were seen for the baseline assessment were offered information on their diagnoses and on professional help options.

### Safety measures

For the protection of the reported data which includes highly sensitive contents, we did not obtain any personal data via the online screening. At the baseline assessment, it was ensured that participants with a history of suicidal ideation had self-help strategies at hand. The diary assessment took place Sunday through Thursday night over two consecutive weeks so that each entry could be checked for occurrence of severe injuries the next morning on a workday. Participants were contacted in case of severe injuries that required medical attention. Additionally, all participants were called twice during the course of the study to keep track of any difficulties.

### Measures

#### Screening

The online screening provided a comprehensive description of NSSI and of NSSI-images and asked about any experience with such images. The exact questions are displayed in Table 1. The scale for dysregulated behaviors of the Borderline Symptom List (BSL-95 [58]) was administered. It assesses 11 dysregulated behaviors such as NSSI, bingeing or high-risk behaviors. This add-on scale has not been included in the assessment of the psychometric properties of the broader measure [58], has however, been used in a study by Cloos et al. [26] who have reported an internal consistency of α = .65. The internal consistency of the scale in our sample was α = .61.

**Table 1:** Questions used in the screening for the assessment of NSSI and NSSI-images

Assessment in the screening	
NSSI	“*Have you self-injured on purpose (*e.g. *by cutting, burning, stinging, beating, chafing) five times or more over the course of the past 12 months without an intention to die?*”
NSSI-images	“*Have you previously experienced any mental images that are for you related to NSSI? How frequently have you experienced those lately?* *E.g., once a week.*”

Open-ended questions were used to assess the frequency of NSSI-images during the previous month.

#### Baseline assessment

##### Demographics

Participants were asked about their age, marital status, ethnicity, and current psychotherapeutic and psychopharmacological treatments in an interview.

##### Clinical diagnoses and risk assessment

All diagnostic instruments were administered to participants by the first author who is a trained psychotherapist; additional measures were administered online.^3^ Both parts of the German version of the structured clinical interview (SKID I and II [56]) were administered to participants. These semistructured interview guides assess any clinical diagnoses of mental disorders in both clinical and research settings [59] based on the DSM-IV [60]^4^. The diagnostic criteria of NSSID (DSM-5) were checked with participants by the first author based on both the participants’ statements and the clinician’s impression. The risk for suicidal and homicidal behaviors was assessed following routine clinical practice.

##### NSSI, NSSI- images and attitude towards NSSI

To assess the NSSI frequency, the scale for dysregulated behaviors of the BSL-95 [58] was administered online, and the item that assesses NSSI frequency during the previous week was used. The overall scale had an internal consistency of α = .35.^5^ We developed the Self-harm Images Interview (SHII) by translating, back-translating and adapting the Suicidal Cognitions and Flashforwards Interview [38] to our research questions. After a comprehensive description of the difference between verbal thoughts and mental images, it explains that images can be of actual NSSI or of any other content that relates to NSSI for the participant, that they can be clear or unclear, and include different sensory impressions (hearing, smelling etc.). All following questions pertain to the most significant NSSI-image. Participants are encouraged to identify this image and to answer open-ended questions such as about the frequency of its occurrence and about its content, about their predominant emotion after the experience, any urges to act, action taken, and the meaning that the occurrence of the image has for them. Rating scales ranging from 1 (e.g., ‘*not at all*’) to 9 (e.g., ‘*extremely*’) are used to quantify important imagery characteristics such as controllability (e.g., ‘*How controllable was the occurrence of the image?*’), compellingness, here-and-now quality, distress or comfort (e.g., ‘*How distressing/comforting was the image?*’) and to assess the person’s attitude towards NSSI. The question regarding the attitude reads as follows: ‘*Some people think that in certain situations it is okay for them to self-harm, others don’t. Please try for a moment not to think of what others might say but what* you *think. Is it okay for you to harm yourself?*’

##### Positive and negative affect

In the SHII we have included the German version of the Positive and Negative Affective Schedule (PANAS [61]). The PANAS has been widely used in clinical assessments. It consists of two 10-item-scales for positive and negative affect with acceptable internal consistencies with a Cronbach’s α of .71 and .77, respectively [61] in our sample, the internal consistencies were α = .58 and .79, respectively. Participants are asked to rate the extent to which each emotion was experienced on a five-point rating scale ranging from 0 (‘*very slightly or not at all’*) to 4 (‘*very much*’). For the study purposes, the timeframe was altered to ‘*immediately after the image*’. The mean of these ten items for positive and for negative affect of the PANAS were used as scores for positive and negative affect after the image respectively in the analyses.

##### Entrapment

The German adapted version of the Entrapment Scale [62] was administered online. Its 16 items are answered on a 5-point-likert-scale from 0-*not at all* to 4-*extremely*. It has an excellent internal consistency (α = .95 [62]); in our sample the internal consistency was α = .88.

#### Daily diary

We created an online short version of the baseline assessment to be completed daily. This included: the PANAS; a single-item question assessing entrapment (‘*How much did you feel entrapped in your current situation today? Meaning that you perceived your current situation as unpleasant (e.g., due to own thoughts or feelings, or to outer circumstances) and would have liked to leave it yet felt unable to.*’) which was answered on a 5-point-likert scale (1-*not at all* to 5-*extremely*); a three-item question on NSSI presence (yes/no), method (using a list of methods of a measure for NSSI from the Deliberate Self-Harm Inventory [63]) and frequency (‘*How many times have you harmed yourself today?*’) as well as a two-item question regarding the time point of NSSI (‘*At approximately what time did you harm yourself?*’, ‘*When in respect to an NSSI-image did you harm yourself?*’), the latter being asked only if participants had reported at least one NSSI-image and being answered by single-choice of the following: *before an NSSI-image*/ *after an NSSI-image*/ *before and after an NSSI-image*/ *unclear or in no timely relation to an NSSI-image*; a two-item question assessing substance use prior to NSSI-image (yes/no) and open-ended specification; a 20-items questionnaire on NSSI-imagery extracted from the SHII and including PANAS affect ratings after experiencing the NSSI-image, *distress*, *compellingness*, *controllability*, *nowness*, *vividness* and *comfort* of images (from 1 = *not at all* to 9 = *extremely*), including also the time point (‘*At approximately what time did the image occur?*’). The internal consistencies for the PANAS were calculated considering the nested structure of the items (i.e., the within-person reliability was calculated according to Cranford et al. [64]), and yielded scores of α = .77 and .84 respectively for positive and negative affect after the image, and α = .92 and .89 for daily positive and negative affect, all measured with the PANAS. The last diary additionally asked about potential bias-effects induced by the diary, such as whether participants believed completing the diary changed the frequency of NSSI and/or of images, reasons for such changes and general adherence to the protocol.

### Data analyses

To assess the rate of people who had experienced NSSI-imagery among those with a history of repetitive NSSI, we calculated the percentage of those who endorsed NSSI-images among our total online screening sample of *N* = 201. Further analyses were calculated using the subsample of *n* = 19 who completed all three parts of the study. To investigate the relationship between NSSI and NSSI-imagery as experienced by individuals meeting the proposed DSM-5 diagnosis of NSSID (subsample of *n* = 19 completers of both the baseline assessment and the daily diary), first we calculated a Pearson correlation between the frequency of NSSI and the frequency of NSSI-images within the baseline data. Next, we compared the respective time points of NSSI and NSSI-images on the 17 NSSI-days to descriptively report the timely relations of both. If the order was indistinguishable (i.e., both were marked with the same time) we additionally checked the participant’s answers regarding the timely relation (NSSI before/after an NSSI-image/both/unclear).

Given that a proportion of participants meeting the criteria for NSSID who completed the interview and daily diary did not engage in NSSI during the observation period, further analyses were carried out using the datasets of the *n* = 10 participants who have self-harmed during the diary. To investigate the relationship between the imagery characteristics and NSSI by considering the nested structure of the data, we first calculated two mean values for each person regarding the imagery characteristics (comfort, distress, controllability, nowness) of NSSI-images, associated affect and entrapment beliefs per person on the accumulated days with (*k* = 17) and without NSSI in the diary (*k* = 172), and then used paired t-tests to compare those means. Considering the nested structure of the data of *k* = 189 entries obtained by *n* = 19 participants, we also tested the variability of the items by calculating the intraclass correlation coefficient (ICC [65]) which indicates the proportion of the between-person variance in the total variance.

Finally, we conducted an exploratory post-hoc analysis of any differences in the presence and characteristics of NSSI-images, associated affect and beliefs between those who engaged in NSSI and those who did not during the diary observation period, by reporting mean values of all variables from the baseline assessment. Since entrapment and comfort had related to NSSI in our diary data, we further explored the association between imagery and entrapment calculating a post-hoc Pearson correlation using the full-length entrapment scale from the baseline assessment.

## Results

### Sample description

#### Screening

The 201 completers of the screening self-identified as female university students who had self-injured at least five times over the course of the previous 12-month-period.

#### Baseline assessment and daily diary

Out of the screening sample of *N* = 201, *n* = 32 were seen for a baseline assessment and diagnostic, and a subsample of *n* = 19 completed all parts (see Fig. 1 for the flow of study participants and Fig. 2 for the flow of procedures). Demographic characteristics of this subsample including information on clinical diagnoses and psychotherapeutic treatment are displayed in Table 2.^6^ One participant reported to receive interventions that directly aimed at mental images or NSSI. All 19 participants completed 10 diary entries except for one who missed the last entry: total diary entries = 189. Entries were delayed to the next morning 13 times (6.87%). Due to the small number of delayed entries, these were included in the total sample.

**Table 2:** Sociodemographic data, diagnoses, and psychotherapeutic treatment

Age, y, (SD)	24.63 (4.52)
Marital status, percentage (n)	
Relationship	57.90 (11)
Married	10.50 (2)
Single	31.60 (6)
Migrant backround, percentage (n)	31.60 (6)
Diagnoses mean number, (SD)	6.05 (2.46)
Diagnoses in addition to NSSID, percentage (n)	
At least one personality disorder	89.47 (17)
Borderline Personality Disorder	78.95 (15)
Affective Disorders	94.73 (18)
Current MDE	47.37 (9)
Past MDE	42.10 (8)
Bipolar Disorder (currently remitted)	5.26 (1)
Psychotherapeutic treatment, percentage (n)	31.58 (6)

Due to the interplay of NSSI(−D) and BPD, further descriptive results in this section will be described both for the sample of *n* = 19 and for the BPD-subgroup of *n* = 15.

### Descriptive data of prevalence of NSSI, NSSI-images and attitude towards NSSI

#### Screening

Of the *N* = 201 female University students who took part in the screening, 168 (83.6%) reported previous experience of NSSI-related images.

#### Baseline assessment

In the baseline assessment, the mean score for NSSI frequency in the scale for dysregulated behaviors of the BSL-95 was .89 times per week (*SD* = .81; individuals diagnosed with BPD *M* = 1.10, *SD* = .80) and the most significant image was reported to have occurred on average 15.32 times during the previous week (*SD =* 39.10; BPD-subsample *M* = 15.20, *SD* = 42.60). Fifteen (78.95%) of our 19 participants rated their most significant NSSI-related image to be of NSSI (78.57% of the BPD-subsample). Those images were most frequently of cutting one’s extremities, yet some were more closely related to suicidal imagery, e.g.:

‘*I see myself as I ( … ) get a large knife with a black blade from steel and I rip, slash, cut open my belly with it. I see the blood flowing and my face that is torn in anger. ( … )*’

Participants who described NSSI-images with contents other than NSSI described (one each) their own funeral after suicide, feared or distressing situations, and more abstract imagery (i.e., intense red color) respectively.

Regarding their attitude towards NSSI, participants appraised harming themselves as moderately ‘*okay*’ with a mean score of 5.11 (*SD* = 3.38; BPD-subsample *M* = 5.13, *SD* = 3.50).

#### Daily diary

Ten of the 19 participants (BPD-subsample: 8 of 15 participants) self-injured between one and three times each (thereof two participants each twice on one day), rendering a total number of 19 NSSI-incidents on 17 days. In two cases, medical treatment was necessary and received. All participants experienced NSSI-images during the observation period. Of the 189 diary entries, experiences of NSSI-images were described on 89 and days (47.10%; see Table 3 below). In terms of contents, images in the diary were categorized by participants as follows: 67.42% NSSI, 12.36% an object related to NSSI (i.e., a razorblade), and 20.22% other (including own suicide). No participant described imagery representing negative impact of NSSI.

One participant reported the use of one glass of alcohol prior to her image experience on one of her 10 days (not followed by NSSI).

##### Prevalence and frequency of NSSI-images

At baseline, our data showed a medium correlation between the frequency of NSSI-images and the frequency of NSSI (*r =* .36, *p* = .13).^7^ Table 3 displays the number of incidents of NSSI and of NSSI-images in the diary.^8^ The sensitivity of images was 0.94, whilst the specificity was 0.42.

**Table 3:** Number (*k*) of days with NSSI and with NSSI-images present in the diary of our *n* = 19 participants

	NSSI-image	no NSSI-image
NSSI	16	1
no NSSI	73	99

##### Timely relation of NSSI and NSSI-images in the diary

Eight people (80%) of the sample of *n* = 10 who self-injured reported that images preceded NSSI-incidents in all cases.^9^ More specifically, on 15 of the 17 days with NSSI-incidents (thus, 88.24%), NSSI was carried out after the experience of at least one NSSI-image and on all of those days at least one NSSI-incident occurred within the first two hours after the experience of an image. Table 4 displays examples of two participants’ descriptions of their images, emotions and meaning, actions afterwards and the respective times of the day on a day on which they afterwards engaged in NSSI and a day on which they did not engage in NSSI.

**Table 4:** Examples of two participant’s diary entries regarding their most significant NSSI-image on days with (left) and without NSSI (right; one participant per row)

Image followed by NSSI							Image followed by different action						
Day in study	Times and number of occurrence of NSSI- images	Content	Predominant emotion afterwards	Meaning of the image	Action after the image	Time of NSSI	Day in study	Times and number of occurrence of NSSI- images	Content	Predominant emotion afterwards	Meaning of the image	Urge to act	Action after the image
4	2 pm (twice)	I see myself as I slit open my arms.	determined	It felt as though the outcome of the fight against the urge to self-injure was decided upon for today.	NSSI	2:30 pm	7	5:15 pm through 9 pm (4 times)	I see my skin, my arms, my upper legs and feel an anxious expectation of pain, I see the traces of bloody scratches on my skin, see my cut nails, imagine the feeling of unfiled edges on my skin	Lost, stupid	Feelings such as self-hatred, shame, stupidity, helplessness, etc. also a defeat before myself and people that I trust, that trust me	Wanted to disappear and to inflict pain on myself because of my existance	cried
4	10 pm-12:30 pm (6times)	I take a knife into my hand. Situation before actual NSSI is displayed. Taking a deep breath. (…) Observer perspective. Feel a tingling, nervousness, excitement, and anxiety. I sweat.	resignation	Pain, satisfaction, anxiety, (…) a task that has yet to be fulfilled today; see the due self-injury as an assignment. Paying for blood as a compensation for overeating (…) NSSI as a way to accept myself.	eating	1 am	3	10 pm-11:30 pm (4 times)	Large knife that I run into my belly. Blood splattering.	Desparate, sad	Rage and hatred towards myself; being torn between the longing to give in to the image and trying to refuse	NSSI	eating

##### Daily diary-induced bias^10^

Eight participants felt that the frequency of images had been unaltered by completing the diary; four participants felt an increase, and six a decrease in NSSI-images relative to their perceived usual. Regarding NSSI, ten participants felt no difference to their usual; four participants reported they had engaged in more NSSI, and four in less NSSI. Six participants who observed changes associated these directly with the study. Moreover, general effects of the diary were described by 13 participants, such as “positive”/“helped me” (4), “distance”/“reflection”/” thoughtfulness” (6), or “trigger”/“more images” (3).

### Characteristics of NSSI-images, affect, and entrapment

The mean ratings of the imagery characteristics at baseline and in the diary as well as the mean scores for positive and negative affect *for the day* (diary) and *after the image* (baseline and diary), the entrapment ratings (baseline and diary) are displayed in Table 5.

**Table 5:** Imagery characteristics, affect ratings, and entrapment at baseline and in the diary

	Baseline			Diary			
	N	M	SD	k	M	SD	ICC^a^
Compellingness	19	6.84	1.77	89	5.77	1.22	0.21
Vividness	19	7.32	2.01	89	7.75	1.29	0.29
Controllability	19	2.37	1.21	89	3.23	1.59	0.22
Nowness	19	4.11	2.81	89	4.42	1.63	0.29
Distress	19	6.89	1.73	89	6.00	1.36	0.33
Comfort	19	2.79	2.64	89	3.03	1.87	0.44
Positive affect after image	19	0.55	0.41	89	0.51	0.43	0.49
Negative affect after image	19	2.15	0.76	89	1.80	0.62	0.43
Positive affect daily	–	–	–	189	.88	0.56	0.47
Negative affect daily	–	–	–	189	.90	0.55	0.41
Entrapment Scale	19	2.01	0.74	–	–	–	–
Entrapment daily	–	–	–	189	2.93	.81	0.37

For the diary data, the within-person reliability was calculated according to Cranford et al. [63]. According to George and Mallery [65], the values are interpreted as excellent if > .9, as good if > .8, as acceptable if > .7, as questionable if > .6, aspoor if > .5, and as unacceptable if < .5

^a^Intraclass correlation coefficient; values below .50 indicate that variance is predominantly due to within-person-fluctuation

The comfort and compellingness of images were higher on NSSI-days compared to days without NSSI, while the controllability and vividness of images were lower on NSSI-days compared to days without NSSI. Daily negative affect was higher and daily positive affect lower while both positive and negative affect *after the image* as well as entrapment beliefs were higher on NSSI-days. Instead, there were no differences in the image nowness and distress. Table 6 displays the mean differences, standard deviations, and statistical comparisons between NSSI-days and other days (i.e., mean scores of each variable on the days with, *k* = 17, and without NSSI, *k* = 172). Note that all effects are non-significant with the exception of a trend regarding controllability (*p* < .10) as signified by the symbol †.

**Table 6:** Mean differences, standard deviation, t-scores (paired), *p*-values, effect size *d* and its interpretation between the accumulated days with (17) and without NSSI (172) of our ten participants who have shown NSSI regarding the imagery characteristics, affect, and entrapment beliefs in the diary

	Mean difference NSSI-days, noNSSI-days (SD)	t	p^a^	d^b^	Interpretation
Compellingness	.90 (1.79)	1.58	.15	.56	Medium
Vividness	−.47 (2.45)	−.61	.56	−.29	Small
Controllability	−1.14 (1.84)	−1.97	.08†	−.89	Large
Nowness	−.03 (1.69)	1.55	.96	−.02	–
Distress	−.16 (1.58)	−.32	.75	−.09	–
Comfort	1.14 (2.33)	1.58	.16	.43	Small
Daily positive affect	−.30 (.41)	−2.26	.05†	.50	Medium
Daily negative affect	.62 (.94)	2.08	.07†	.74	Medium
Positive affect after image	.17 (.68)	.78	.45	.24	Small
Negative affect after image	.26 (.90)	.90	.39	.28	Small
Entrapment	.51 (1.13)	1.42	.19	.47	Small

^a^level of significance: †: *p* < .10

^b^regarding the effect size, we first obtained the d_z_ for paired t-tests which, however, overestimates the effect size compared to independent groups and should therefore be adjusted (e.g., [67]), and therefore obtained the d_av_ according to Cumming [68]

### Exploratory results

Table 7 displays differences in the presence and characteristics of NSSI-images, associated affect and beliefs at baseline between those who had and those who had not engaged in NSSI during the dairy observation. Additionally, there was a strong and significant positive correlation between the score for entrapment and the comfort associated with the NSSI-image as assessed at baseline (*r* = .64, *p* = .00).

**Table 7:** Group means and differences of participants who did (“NSSI”) and did not show NSSI (“noNSSI”) regarding the imagery characteristics, affect, entrapment belief and attitude towards NSSI at baseline

	M (SD)		Mean difference
	NSSI	noNSSI	
Compellingness	6.60 (1.58)	7.11 (2.03)	−0.51
Vividness	6.90 (2.28)	7.78 (1.79)	−0.88
Controllability	2.50 (1.35)	2.22 (1.09)	0.28
Nowness	3.20 (2.20)	5.11 (3.18)	−1.91
Distress	7.00 (1.89)	6.78 (1.64)	.22
Comfort	3.30 (3.29)	2.22 (1.72)	1.08
Neg.affect after image	1.95 (.77)	2.37 (.73)	−0.42
Pos.affect after image	.57 (.43)	.53 (.41)	0.04
Entrapment	2.19 (.79)	1.82 (0.66)	0.37
Attitude towards NSSI	5.40 (3.41)	4.78 (3.53)	.62

## Discussion

First, among our large sample of 201 screening participants, a majority of approximately 84% experienced NSSI-images. This finding is similar to previous online survey data in student samples [37] and confirms the role of NSSI-imagery as a common phenomenon among young people engaging in this behavior.

Second, our study was the first to: 1) assess imagery in a transdiagnostic, clinically quite severe sample with a mean of six diagnoses each including at least one personality disorder (except for two participants); and 2) use a daily diary rather than retrospective measures. In terms of imagery presence, we found that the frequencies of NSSI and NSSI-images were medium correlated at baseline assessment; and that during the observation period of the diary, NSSI-images were always, except for once, present when NSSI was carried out (NSSI-images appeared as highly sensitive yet not very specific indicators of NSSI), and the images were reported to have occurred first (with two exceptions); this supports our first hypothesis on the relationship between images and NSSI and adds that in this sample, images preceded NSSI in almost all cases. The results stress the importance of considering mental images when assessing and treating NSSI. Roughly 80% of NSSI-images were indicated as being of actual NSSI or of an object associated with NSSI (i.e., a razorblade). Contrary to the finding of McEvoy et al. [37], no participant in our study described image(s) of negative outcomes of NSSI. Since the instruction was to describe only the most significant image, if any such images had occurred, they seemed not to have been perceived as most significant, even on days when participants had not engaged in NSSI.

In terms of imagery characteristics, we observed that in the baseline assessment, images were perceived by our participants twice more distressing than comforting and were characterized by a rather low overall controllability. The differences between NSSI-days and other days in the diary pertaining to our hypotheses were all non-significant, and are interpreted based on the effect sizes which were small to large according to Cohen [66]; this decision was based on research that points to the relevance of effect sizes as reliable indicators of differences (e.g., [69]). During the diary observation, on NSSI-days images were perceived as particularly comforting yet also particularly intrusive and compelling. This variety of appraisals of imagery extends the notion that while some imagery characteristics such as intrusiveness appear to be transdiagnostically associated with symptom severity [24], other characteristics may tend to vary by psychopathology such as, e.g., compellingness in Bipolar Disorder [70], or the perspective of images in Social Phobia (e.g., [71]). Our findings only partly confirmed our hypotheses around imagery characteristics (note that vividness was rated lesser on NSSI-days in our data), possibly also due to the diversity and complexity of underlying psychopathology in our sample. As NSSI is a transdiagnostic phenomenon, future studies may investigate whether NSSI-images differ depending on the presence and nature of comorbid conditions.

Imagery comfort had been previously related to the severity of suicidality [72], and although NSSI-images were generally perceived as quite distressing, only high imagery comfort marked NSSI-days within our data. It is possible that a shift occurs from distress to comfort in the appraisal of NSSI-images (perhaps in relation to entrapment, see below) marking greater volitional impact of the images towards engaging in the behavior. The comfort associated with NSSI-images may represent positive (soothing) appraisals of NSSI that can maintain a disorder such as NSSID [6]. Previous findings of imagery distress being negatively associated with the severity of suicidality [72] could thus not be replicated with regards to NSSI within our data.

Entrapment beliefs were increased on NSSI-days compared to days without NSSI which confirms that entrapment may be relevant in NSSI as well as in suicidality (e.g., [31]). Entrapment beliefs should be a focus in the treatment of individuals who wish to reduce NSSI. As self-efficacy with respect to more adaptive coping mechanisms could help prevent engagement in NSSI [48], enhancing self-efficacy in those with high levels of entrapment could represent an important treatment target.

Finally, the reported increase in both positive and negative affect after the NSSI-image may indicate that NSSI-images can be quite ambivalently valanced experiences, e.g., mirroring expectations of an emotional relief [37] and wishes to refrain from the behavior as verbalized by many of our participants. However, on NSSI-days compared to days without NSSI, negative affect was increased, and positive affect was decreased at night which may indicate that mood dropped following an incident of NSSI. An increase of negative emotionality in the aftermath of NSSI has previously been reported by Houben et al. [53] when administering ESM (Experience Sampling Method) to a sample of patients with borderline personality disorder. These findings may suggest that although NSSI is often carried out with the expectation of an emotional relief (e.g., [6, 37]), this relief may be short-lived. More research into the course of emotionality in NSSI and the role of imagery using ESM is necessary to further our understanding of NSSI.

Our exploratory post-hoc analyses support the findings mentioned above, albeit tentative. Participants who have shown NSSI displayed a more positive attitude of finding it ‘ok to self-harm’, less negative affect after the image, and more comforting appraisals of the image. Entrapment beliefs were also higher in this group and overall entrapment beliefs appeared to correlate strongly with the image comfort. If replicated in a larger sample, this may indicate that situations of blocked escape facilitate the use of self-damaging behaviors such as NSSI, and the relief from entrapment is encapsulated in comforting imagery, similar to what was previously reported for suicidal imagery [31].

### Limitations

Our first limitation is the sample size of the NSSID group, such that the comparisons between those who did and did not engage in NSSI over the diary observation period remain preliminary, exploratory and descriptive. Further, the rate of NSSI in the two-week observation period was quite low with little more than 50% in a sample that had been selected based on their regularity of NSSI. One can easily assume a study intervention effect as 53% of participants reported they had observed deviations from a regular period and as self-monitoring has been found effective in modulating behavior (e.g., [73]). To address the issue of a potential study intervention effect, a more anonymous setting could be beneficial such as changing the order of the diary and the interview and diagnostic session (this could result in a less economical way of data collection as people may have to be excluded after participation if they meet exclusion criteria), or by the employment of more research assistants, as all parts of the study were conducted by the same researcher and the participants may have bonded with her. The study might be conducted online without any previous contact to the researchers which would, however, result in a reduced external validity due to lack of clinical diagnoses, and in less secure ways of data collection, and possibly higher abortion rates. We conclude that more satisfactory changes to methodology are still needed.

In terms of statistics, conducting multilevel modelling would have been desirable. However, since only some participants engaged in NSSI in an already small sample, most data points would not have been in the analysis and the sample size requirements for this method were thus violated. In addition, we found mostly small to medium effects; both the small sample size/low rate of NSSI and the smaller-than-expected effect size reduced the power and caused deviations from our a priori power calculation. The reported results from the group comparisons remained nonsignificant. Similarly, Ammerman et al. [74] reported that when administering ESM to a sample of *N* = 51 patients with BPD over the course of 1 week, only 26% of the sample self-injured. Associated with the low rate of NSSI during the observation period, another limitation is the large difference in the number of days with (*k* = 17) and without NSSI (*k* = 172).

Moreover, our assessment of the frequency of NSSI during the diary could be imprecise as our participants who have reported an NSSI-incident during the diary may have referred to a session with one cut/burn etc. or a session with several cuts/burns/ etc.

The generalizability of our findings is further limited as we report data from a specific sample of female university students, and we specifically selected for NSSID so that some features may not translate to NSSI outside NSSID. The exclusion of male participants is a limitation because, although research suggests NSSI can vary significantly by sex (e.g., [55]), recent evidence found similar NSSI characteristics in both sexes in a community sample [75]. Moreover, effects may also partly pertain to the symptoms of other mental disorders as almost all participants had at least a history of depressive disorders, and as 79% were diagnosed with Borderline Personality Disorder (BPD).

Although our imagery assessment can be said to meet the gold standard in this field of research [24], no statement can be made about the validity of the mental imagery questionnaire beyond face validity. The same is true for the entrapment rating in the diary which was assessed using a single question for ecological reasons. Although single-item assessments are common in ESM studies [76], they also hamper the reliability of the findings. As the NSSID-diagnosis is still preliminary [6], a possible future alteration of the NSSID-criteria may retroactively affect the validity of our findings. The reliability of the DBS scale was with α = .35 unacceptably low [77]. Please note, however, that for our analyses, only the item on NSSI-frequency has been used, and that the low alpha coefficient may be due to the nature of the scale, i.e., a checklist of behaviors, rather than to a low reliability (e.g., [78]).

### Implications

If replicated in larger samples, our data support the notion that imagery can be an important element in the processes that drive maladaptive behavior [35], especially when perceived as intrusive and in personal situations marked by entrapment and negative affect. Engaging in NSSI even on the cognitive level could increase the future probability to act upon it [79], and simulating an action can make it more likely to engage in the action [80]. However, the imagination of a desired outcome such as a calm and relieved emotional state could also lead to a declined motivation to act on an urge including NSSI, which has been shown for adaptive goal-directed behaviours [81]. An NSSI-image that is purposefully evoked (rather than intrusive) could at least in the short run serve as a substitute for carrying out NSSI yet still contribute to the maintenance of the behavior in a longer time span. NSSI assessments in clinical practice should include imagery and idiosyncratic formulation of its role for each individual patient.

If our data are replicated in a larger sample, several implications may be drawn for the treatment of NSSI and NSSI-images. Grounded in mindfulness and acceptance, practitioners could proactively address the high ambivalence or even positive appraisals around NSSI and NSSI-images. They could help patients change the outcome of NSSI-images as suggested by Holmes and colleagues regarding suicidal imagery [39] or use meta-cognitive techniques that increase the sense of control over the image [82]. The entrapment beliefs could be analyzed, and help provided for people to regain a sense of control and self-efficacy. Future studies should aim to fully understand the complex interactions between imagery characteristics, beliefs and affect that may or may not facilitate NSSI, for example via closer observation intervals administered to participants several times per day, for longer observation periods and including direct imagery / affect manipulations. Finally, as our data is only correlational, multilevel modeling and regression analyses would also be desirable on larger samples (with desirably more similar numbers of days with and without NSSI) to draw more precise conclusions on the causal nature of imagery and derive more specific intervention methods from the data.

## Conclusions

Data from our sample of *N* = 201 students who reported repetitive engagement in NSSI confirm that NSSI-images may be a common phenomenon in this population. The interview and diary data (*n* = 19) further preliminarily indicate NSSI-images may capture ambivalent (positive and negative) appraisals of NSSI; positive (soothing) appraisals and emotions around NSSI-images appeared more salient to people when NSSI was carried out. NSSI-images were a sensitive correlate of NSSI and usually preceded NSSI in time; they should thus be a focus in the psychotherapeutic treatment of people who seek help in reducing this behavior or can even be diagnosed with a disorder such as NSSID. The study provides information on feasibility and methodological challenges such as intervention effects of the diary. More research using larger samples and hierarchical modeling is needed to confirm the results.

## Data Availability

The datasets used and/or analysed during the current study are available from the corresponding author on reasonable request.

## Abbreviations

BPD: Borderline Personality Disorder
BSL-95: Borderline Symptom List, 95-item-version
DSM: Diagnostic Statistical Manual of Mental Disorders
ESM: Experience Sampling Method
ICC: Intraclass Correlation Coefficient
ICD: International Classification of Disorders
MDE: Major Depressive Episode
NSSI: Nonsuicidal self-injury
NSSID: Nonsuicidal self-injury disorder
PANAS: Positive and Negative Affective Schedule
SD: Standard deviation
SHII: Self-Harm Images Interview
SKID: Strukturiertes Klinisches Interview für DSM-IV

## Footnotes

1 For the diagnostic criteria of NSSID see section Procedure and sample selection.

2 All compensations and incentives were funded internally.

3 No interrater reliability can be established since all diagnostic measures were applied by the first author.

4 These are currently the only in German available interviews based on the DSM.

5 Please note that the scale is not used for further analyses in this study.

6 For data safety regarding a quite sensitive topic and to facilitate participation of a large sample, we did not assess any personal data online and can thus make no statements about demographics of this sample.

7 Interpretations of correlations and effect sizes according to Cohen [66].

8 Please note that two participants reported two NSSI-incidents on 1 day each.

9 One person said images had preceded NSSI in one of two cases and one person reported not to remember the time of the image.

10 Please note that since one participant missed the last diary entry in which this information was obtained, we report data of the remaining *n* = 18 completers of the diary.
